# Accessing the biocompatibility of layered double hydroxide by intramuscular implantation: histological and microcirculation evaluation

**DOI:** 10.1038/srep30547

**Published:** 2016-08-02

**Authors:** Vanessa Roberta Rodrigues Cunha, Rodrigo Barbosa de Souza, Ana Maria Cristina Rebello Pinto da Fonseca Martins, Ivan Hong Jun Koh, Vera Regina Leopoldo Constantino

**Affiliations:** 1 Departamento de Química Fundamental, Instituto de Química, Universidade de São Paulo-USP, Av. Prof. Lineu Prestes 748, CEP 05508-000, São Paulo, SP, Brazil; 2 Departamento de Morfologia e Genética, Universidade Federal de São Paulo–UNIFESP, Rua Botucatu 740, CEP 04023-900, São Paulo, SP, Brazil; 3 Instituto Biológico, Secretaria da Agricultura e Abastecimento, Av. Conselheiro Rodrigues Alves 1252, CEP 04014-002, São Paulo, SP, Brazil; 4 Departamento de Cirurgia, Universidade Federal de São Paulo-UNIFESP, Rua Botucatu 740, CEP 04023-900, São Paulo, SP, Brazil

## Abstract

Biocompatibility of layered double hydroxides (LDHs), also known as hydrotalcite-like materials or double metal hydroxides, was investigated by *in vivo* assays *via* intramuscular tablets implantation in rat abdominal wall. The tablets were composed by chloride ions intercalated into LDH of magnesium/aluminum (Mg_2_Al-Cl) and zinc/aluminum (Zn_2_Al-Cl). The antigenicity and tissue integration capacity of LDHs were assessed histologically after 7 and 28 days post-implantation. No fibrous capsule nearby the LDH was noticed for both materials as well any sign of inflammatory reactions. Sidestream Dark Field imaging, used to monitor in real time the microcirculation in tissues, revealed overall integrity of the microcirculatory network neighboring the tablets, with no blood flow obstruction, bleeding and/or increasing of leukocyte endothelial adhesion. After 28 days Mg_2_Al-Cl promoted multiple collagen invaginations (mostly collagen type-I) among its fragments while Zn_2_Al-Cl induced predominantly collagen type–III. This work supports previous results in the literature about LDHs compatibility with living matter, endorsing them as functional materials for biomedical applications.

Two-dimensionally organized (2D) materials have been explored for application in tissue engineering and other fields of biomedical interest such as drug delivery and imaging^1^. Layered Double Hydroxides (LDHs) are 2D materials recognized as potential resources for therapeutic and imaging purposes owing to their biocompatibility and low toxicity, among other properties essential to nanomedicine^2^,^3^. LDHs have a general formula [M^II^_(1-x)_M^III^_x_(OH)_2_]A_x/*m*_.nH_2_O, wherein M^II^ and M^III^ are divalent and trivalent cations, and A is an anion of valence *m* which occupies the interlayer region (simplified writing: M^II^_R_M^III^-A with R equal M^II^/M^III^ molar ratio)^2^. Metal cations such as Mg^2+^, Zn^2+^, Al^3+^, and Fe^3+^ fill the center of octahedral [M(OH)_6_] units sharing the edges, which produce a sheet-like frame, as illustrated in Fig. 1. The positively charged LDH sheets stacked face to face resulting in layers with charge deficiency. Anions are presented in the interlayer region to maintain the charge balance and may be exchanged by several organic anions, including drug molecules or genetic materials, resulting in diverse health care applications^1^,^2^,^3^,^4^,^5^,^6^,^7^,^8^,^9^,^10^,^11^.

The biocompatibility of LDH has been explored by *in vitro* tests such as cell viability tests^12^,^13^, lactate dehydrogenase leakage assay^12^,^13^,^14^,^15^, inflammatory mediators analysis^12^, hemolysis assay^15^,^16^,^17^, thrombosis assay^17^, lipid peroxidation^18^, reactive oxygen species (ROS) generation^12^,^14^ and apoptosis^12^,^14^. On the other hand, the *in vivo* biocompatibility and the therapeutic potential of LDH particles have been investigated by a minor number of assays through the analysis of biodistribution and accumulation in tissues^18^,^19^,^20^,^21^, stomach lesions^22^, therapeutic effects as anti-tumor^23^ and anti-inflammatory activities^16^. Latterly, interesting reports have explored the usage of LDH as a grout material in bone cement for implant in tibia^24^, and as a coating to middle ear prostheses^25^. Both studies confirmed that the implants are biocompatible and their performances are enhanced by the LDH presence. A recent work using embryonic stem cells has demonstrated the possibility of employing LDH in regenerative medicine^14^.

Materials biocompatibility can be accessed by methods such as cell cytotoxicity, genotoxicity (mutagenic potential), hemolysis, pyrogen test and intramuscular or subcutaneous implantation^26^. As compared to *in vitro* tests performed in controlled conditions, the *in vivo* intramuscular implant test allows the activation of full biological host defense by the immune response, being in our opinion a far more adequate test to scrutinize, in short and long periods of observation, the materials biocompatibility and functionality aiming tissue engineering or drug delivery applications. Intramuscular implant is an appropriate assay once the muscle is a vascularized region, located far from vital organs, thus being suitable for screening local toxicities by inflammatory response in a short period (7 days) and the rejection process by fibrous capsule formation nearby the implanted material in a longer period (28 days).

Although the biocompatibility of LDH has been explored by *in vitro* and *in vivo* tests as reported in the works already described in this paper, it is observed a lack of studies about intramuscular implantation in order to access the biocompatibility of this class of nanoparticles.

Histological analysis of organs such as liver and kidney of animals have been performed to establish the LDH biocompatibility when administered as drug carrier orally^18^ or injected intraperitoneally^21^. Histology of subcutaneous tissue was also used to evaluate the biocompatibility of an organic hydrogel containing LDH and an anti-inflammatory aiming the intradiscal administration for intervertebral disc degeneration^27^. In this work, histologic studies were performed to access the biocompatibility, biointegration and antigenicity aspects of LDHs intramuscularly implanted, as well, evaluate the modulation and the collagen-type formed in the tissue repair. It is emphasized that no drugs or bioactive species were intercalated between LDH layers.

Additionally, the Sidestream Dark Field (SDF) imaging, a stroboscopic LED ring-based videomicroscopy^28^,^29^, was used to monitor in real time the microcirculation pattern of the tissues around LDH tablets implantation. The presence of the microcirculatory injury is one of the first signs of a local inflammatory event, probably attributed to the antigenicity of the implanted material. This technique was exploited for the first time to investigate the biocompatibility of nanoparticles through the microcirculation inspection.

## Materials and Methods

Magnesium chloride hexahydrate (MgCl_2_·6H_2_O, Synth), zinc chloride (ZnCl_2_, Aldrich), aluminum chloride hexahydrate (AlCl_3_·6H_2_O, Aldrich), sodium hydroxide (NaOH, Merck), paraformaldehyde solution 4% (Synth), historesin (Technovitz^®^7100, Kulzer), hematoxylin (C_16_H_14_O_6,_ Vetec), eosin (C_20_H_6_Br_4_Na_2_O_5_, Vetec), Picrosirius red (C_45_H_26_N_10_Na_6_O_21_S_6_, Alfa Aesar), and polypropylene mesh (Prolene^TM^, Ethicon) were used without further purification or treatment. ICP emission spectroscopy (ICP-AES) analysis was performed in duplicate on an equipment Spectro Analytical Instruments at the Instituto de Química (Universidade de São Paulo-USP). X-ray diffraction (XRD) patterns of powdered samples were recorded on a Rigaku diffractometer, model Miniflex, using CuK*α* radiation (1.5418 Å, 30 kV, 15 mA, scan range 1.5–70°/2*θ,* scan step of 0.03°/2*θ*) and Ni filter. Particle size and Zeta potential were measured in Zetasizer NanoZS of Malvern Instruments. Mass coupled thermal analyses (TG-DSC-MS) were performed on a Netzsch thermoanalyser model TGA/DSC 490 PC Luxx coupled to an Aëolos 403 C mass spectrometer, using a heating rate of 10 °C/min and under synthetic air flow of 50 mL/min. Fourier transform infrared (FT-IR) spectra of samples diluted in KBr were recorded in the 4000–400 cm^−1^ range on a Bomen spectrophotometer, model MB-102, with a coupled diffuse reflectance accessory (Pike Technologies, Inc.). Fourier transform Raman (FT-Raman) spectra were recorded in a FT-Raman Bruker FRS-100/S spectrometer using 1064 nm exciting radiation (Nd:YAG laser Coherent Compass 1064-500 N) and a Ge detector.

Adult female Wistar rats were purchased from UNIFESP animal colony (CEDEME) and kept in adequate environment, fed with proper food pellets and water *ad libitum*. International guidelines for the care and use of animals were followed and the experimental protocol was approved by the Local Ethical Committee (CEUA N° 873141013).

### Layered Double Hydroxides Synthesis

The LDHs (Mg/Al or Zn/Al) containing the anion chloride was prepared by the co-precipitation method^30^. The di- and trivalent metal cations (M^2+^/M^3+^ molar ratio equal to 2) solution was added into the deionized water adjusted previously with the solution of NaOH (0.2 mol L^−1^) at the pH of the particular LDH composition (pH 9–10 for Mg/Al and pH 8–9 for Zn/Al samples). The addition of the metal cations solution was completed under nitrogen atmosphere with vigorous stirring. The obtained suspension was aged at 25 °C for 1 h under N_2_ atmosphere and then washed with deionized water by filtration under reduced pressure. The isolated solids were dried at room temperature for 48 h under reduced pressure, and were abbreviated as Mg_2_Al-Cl and Zn_2_Al-Cl. Chemical analysis of Mg_2_Al-Cl sample: molar ratio Mg/Al = 2.1; wt.% H_2_O = 16.2. Chemical analysis of Zn_2_Al-Cl sample: molar ratio Zn/Al = 2.08; wt.% H_2_O = 9.1.

### Intramuscular Implantation assessment

The *in vivo* biocompatibility assays were performed by the implant of heat sterilized tablets (5 mm of diameter x 2 mm of thickness) containing compacted particles of Mg_2_Al-Cl and Zn_2_Al-Cl samples. Rats weighing 200 to 235 g were subjected to ketamine and xylazine (4:1) anesthesia (0.1 mL/100 g of body weight, intramuscular). Following midline skin incision, subcutaneous space was dissected laterally, and LDH tablet was implanted into the space between external and internal muscle layer, created by small incision over the external oblique muscle and blunt dissection between two muscle layers. The same procedure was carried out at the opposite side in order to compare Mg_2_Al-Cl and Zn_2_Al-Cl samples biocompatibility in the same animal. The peritoneal cavity remained intact without perforation. In the control, the surgical procedure used was similar to other groups, but without the LDH-tablet implantation (sham-operated animals), in order to check out the sole effect of the surgical trauma involved in this process (n = 5). Thereby, the judgment of the LDHs tablets plus the surgical trauma effect on the host response could be better analyzed. Moreover, for comparison purpose, a polypropylene surgical mesh with recognized antigenicity was implanted in the midline of the rat abdomen (2 cm × 3 cm) and fixed with polypropylene suture 4/0. After 90 days, their biocompatibility was assessed by histology.

The skin closure of all animals was done with 4/0 continuous nylon suture. All surgical procedures were implemented under aseptic conditions. The tissues holding the tablets were collected after 7 and 28 days after implantation under the same general anesthesia. There were analyzed histological materials of five animals after each post- surgical time (n = 5/period). The host biological responses to LDH tablets were compared to those animals subjected to polypropylene mesh implantation in the abdominal ventral hernia (n = 5).

### Sidestream Dark Field

At post-operative periods, before the tissue sample collection containing LDH tablets, the microcirculatory hemodynamic images of muscular tissues, around and over the tablets, were captured by SDF-gun illuminating the tissue with polarized green pulsed light (LED) while the rats were under anesthesia. The real time images were recorded and further analyzed by AVA-3.1 software. The same procedure was carried out in animals subjected to surgical manipulation only (sham group).

### Histological Assessment

The histological assays of the tissues were performed 7 and 28 days after implantation of LDHs tablets or surgical manipulation only. The collected sample was fixed in paraformaldehyde solution (4%) in PBS 0.1 mol L^−1^, pH 7.4. After serial dehydrated process in a solution of ethanol from 50 to 100% for 10 min, the samples were kept for about 12 h (overnight) in absolute ethanol and historesin (1:1). The samples were immersed in pure resin for 4 h and subsequently embedded in resin to polymerize in appropriate plastic molds. Four histologic sections of 2 μm thickness of each sample were stained by Hematoxylin-eosin and Picrosirius red staining technique. The histological analyses were performed under light microscope Carl-Zeiss Axio Scope A1^®^.

## Results

### Materials Characterization

LDH samples characterization by chemical analysis, XRD, vibrational spectroscopy and thermal analysis confirm the isolation of layer structured materials with the following compositions: [Mg_2.10_Al(OH)_6.20_]Cl∙2.3H_2_O and [Zn_2.08_Al(OH)_6.16_]Cl∙1.7H_2_O. The medium hydrodynamic diameter of about 80–100 nm observed for LDHs samples is considered ideal to preclude nonspecific capture by macrophages of reticuloendothelial system, for long-standing circulating and delivery system^31^. Materials characterization data (Figures S1–S3) and the related interpretation are shown in details in Supplementary Information.

### Macroscopy, Histology and Sidestream Dark Field Assessment

The first indication of biocompatibility of Mg_2_Al and Zn_2_Al samples was the absence of signs of inflammation in the site where the tablets were implanted such as edema, erythema and increase in tissue volume, confirmed by the macroscopic inspection (Fig. 2). These findings on the 7th postoperative day (period defined as the acute phase of the inflammatory response) suggest a possible biocompatibility of the LDH samples (Fig. 2A). Besides, the persistence of non-inflammatory reaction around the implants at 28 days (Fig. 2B) strongly indicates that both LDH tablets are not antigenic even in a prolonged period.

To corroborate the macroscopic inspection, microcirculatory assay was performed in muscle tissues where the tablets were implanted in order to evaluate the LDH effects towards the local microcirculation. The presence of persistent inflammation by foreign body antigenic contents (antigen) can compromise the local microcirculatory integrity by being subject to pro-inflammatory mediators. Examination by video microscopy (SDF) revealed an overall integrity of the microcirculatory network in muscle tissues involving the tablets (Fig. 2A,B). The continuous flow of red blood cells in the images can be visualized in the Videos S1–S4 available in Supplementary Information. No obstruction, bleeding and/or increasing of leukocyte-endothelial adhesion were noticed. Such factors are normally present in microvessels exposed to nearby antigenic material implants. These results clearly imply a probable feature of biocompatibility of the LDH once no microcirculatory dysfunction was observed around the tablets. These results were similar to the sham control group (Fig. 2C and Videos S5–S6), which received identical surgical procedures as the experimental group but without deploying tablets; no alterations were observed in the microcirculatory pattern. Furthermore, the antigenicity and tissue integration capacity of LDH samples were assessed through histologic assays at 7 days (Fig. 3) and 28 days post-implantation (Fig. 4) to evaluate a full course of an inflammatory response, stimulated by LDH presence associated with muscle injury owing to the trauma caused from surgical implantation of the tablets.

The histological analysis after 7 days of Mg_2_Al-Cl tablet implantation indicated a benign inflammatory response, suggesting a physiological repair response following a mild surgical tissue trauma and without antigenic stimulus of the implanted foreign body (Fig. 3, superior row). The tablet interface and the surrounding tissue showed occasional neutrophils combined with a cellular organization comprising mainly of fibroblasts, fibrocytes (an inactive mesenchymal cell), macrophages and neovessels. Although similar cellularity was observed in the interface of Zn_2_Al-Cl tablet (Fig. 3, middle row), a thicker layer of cells with a larger number of neovascularization was noticed when compared to the magnesium LDH. In general, the cellular response at 7^th^ postoperative day matches with the physiological tissue repair without signs of an inflammatory response to an antigenic material. In the sham group, histological pattern found in the 7th P.O. day was of normal aspect (Fig. 3, inferior row) even having been carried out the dissection of the space between the abdominal muscles to mimic the introduction of a tablet.

After 28 days, that conclude the final cycle of the acute tissue repair, LDH tablets of both compositions showed no antigenic signs, denoting an excellent biocompatibility when implanted between the muscle layers of the abdominal wall (Fig. 4, superior and middle rows). The tablet as well the tissue interface with both LDHs demonstrated the tissue reconstruction with numerous newly formed microvessels. Moreover, it was observed fibroplasia perfectly organized in layers, the presence of neoformed collagen and the absence of fibrosis in the boundary tablets. These repair features can be correlated to effective tissue integration without chronic inflammation, characteristic of foreign body biocompatibility within a complete tissue repair cycle of 28 days (Fig. 4, superior and middle rows).

Slides prepared after 28 postoperative days were posteriorly stained with red Picrosirius to evaluate the collagen remodeling and type surrounding the implanted tablets. The newly tissue formed between the muscle and the LDH tablets showed the predominance of collagen fibers (Fig. 4, right column). The absence of neutrophils, macrophages, lymphocytes and the presence of a very few quantity of granulation tissue around both LDH implants indicate a physiological pattern of tissue repair. The distinction between Mg_2_Al-Cl and Zn_2_Al-Cl tablets was the thin connective tissue that surrounds the magnesium sample and the clear presence of numerous invaginations of collagen fibers between the spaces resulting from the fragmentation of Mg_2_Al-Cl tablet (Fig. 4, superior row). In the assays with the zinc LDH, it is noticed only the beginning of local collagen fibers invaginations to the tablet core, and the presence of more thickened collagen fibers surrounding the tablet (Fig. 4, middle row).

The assessment of both LDH samples with polarized light showed a different pattern in the collagen composition. The newly formed connective tissue around Mg_2_Al-Cl tablets had a weak birefringence (green/red color), but with predominance of red color suggestive of collagen type-I (Fig. 4, superior row, white arrowheads). In addition, the collagen deposited around Zn_2_Al-Cl tablet showed low birefringence, with a predominance of green color, suggestive of collagen type-III (Fig. 4, middle row, green arrowheads). In the control group (sham surgery), histological pattern found in the 28th postoperative day (Fig. 4, inferior row) continued to be of normal aspect, and the collagen, between the abdominal muscles, was of the prevalence of type-I based on the strong birefringence.

Furthermore, the most commonly used polypropylene surgical mesh were implanted in abdominal wall and the histological data of the ninety postoperative day were added for the comparison purpose in order to illustrate the host signs of rejection when there is an antigenic material implanted in the abdominal wall (Fig. 5). The polypropylene mesh provoked a chronic granulomatous inflammatory reaction around mesh-fibers with the predominance of macrophages, epithelioid cells, giant cells, lymphocytes and mast cells. Areas of tissue necrosis with the predominance of neutrophils and cellular debris were noticed (Fig. 5, superior and middle), which indicated a pattern of persistent acute inflammatory reaction due to the material antigenicity. The collagen deposition was almost absent around the mesh-fibers (Fig. 5, inferior, circles indicated by the capital letter “P”) suggesting a dysfunctional pattern of the tissue reconstruction around the polypropylene mesh.

## Discussion

The detailed discussion about the materials characterization is shown in Supplementary Information. It is worth to emphasize that the LDHs in the form of chloride were chosen since they are frequently taken as precursors for the preparation of LDH intercalated with drugs by ion exchange method. When using co-precipitation method for LDH–drug synthesis, metal cations in the form of chloride salts are used as reagents and Cl^−^ can be co-intercalated with drugs, as observed for pravastatin and mefenamate LDH carrier^16^,^32^.

SDF technique allows evaluate the functional state of the microcirculation through the analysis of the red blood cell flow in the capillaries. The experiment comprising images (Fig. 2) and video (Videos S1–S4, Supplementary Information) of the tissue where LDH tablets were implanted strongly suggest a probable feature of the biocompatibility of inorganic material. According to histological study, LDHs cause no cytotoxicity for the local tissues around the tablets and neither an inflammatory response related to the materials antigenicity. Moreover, they allow a natural tissue integration with functional neovessels and without local microcirculatory dysfunction. To the best of our knowledge, this is the first report about the usage of SDF imaging to access indirectly the biocompatibility of materials implanted in living tissues.

The excellent wound healing around the tablets evokes that LDHs are someway modulating the inflammatory response. Some parameters such as the chemical composition of the material, their acid-base properties and the particles Zeta potential were considered to rationalize the results obtained by histology and SDF image. Other parameters can be relevant as demonstrated in a study about the *in vitro* immunological activities of diverse cultures of LDHs^33^. The immune response of the human monocyte-derived dendritic cells correlated with the follow LDHs physicochemical properties: ionic radius of monovalent and divalent metal cations, interlayer spacing, and Zeta potential.

In this work, the metal cations activities in the living organism were considered. Studies have shown that magnesium has vasodilation action, reducing vascular resistance and increasing the blood flow^34^. Magnesium chloride salt exhibits anti-inflammatory effect by reducing leukocyte migration, Tumor Necrosis Factor (TNF-α)^35^, whereas magnesium sulfate reduces platelet aggregation, TNF-α and Interleukin 6 (IL-6)^36^,^37^,^38^. Zinc chloride presents proliferative action by stimulating Phosphatidylinositol 3-kinase-protein kinase B (PI3K-AKT) pathways, P44/42 Mitogen-Activated Protein Kinases (MAPK), JNK/SAPK (c-Jun-amino-terminal Kinases/Stress-activated Kinase) signal and mammalian Target of Rapamycin (mTOR)^39^. In addition, zinc ions exhibit antioxidant and anti-inflammatory activities by decreasing ROS generation and down-regulating TNF-α and IL-1β^40^,^41^. Additionally, zinc ions stimulate neovascularization by activating growth factors Vascular Endothelial Growth Factor (VEGF), Insulin Growth Factor 1 (IGF-1) and Transforming Growth Factor beta-1 (TGF-β1)^42^. These properties seem to justify such tissue repair and extracellular matrix deposition around LDH tablets here analyzed.

In the present work, it is observed different behavior in the repair process according to the tablets composition, which can be due to the biological response to the different metal cations present in LDH structures. Both tablets show one metal (Al^3+^) and anions (Cl^−^ and OH^−^) in common, and two different cations (Mg^2+^ and Zn^2+^). Interactions between specific salt-protein regulate processes such as the macromolecule folding, association, stability, and precipitation^43^. Kosmotrope ions established stronger charge-dipole interactions with local water than chaotropes ions, which yield distinct modifications in the protein environment, promoting different processes. A protein structure/assembly can be influenced by the nature of anions and cations of a solid surface^44^. Considering the specific ion effects in biological systems and the ions present in the LDH structures here investigated, the cation efficiency as salting-out agent for example is Mg^2+^ > Zn^2+^ > Al^3+^ (kosmotropes), while the anion efficiency is OH^−^ (kosmotrope) >Cl^−^ (borderline)^43^.

The histological difference observed regarding the collagen type formed in the tablets surroundings (Fig. 4, right column) appears to be due to the presence of distinct cations (Mg^2+^ or Zn^2+^). Study performed about pancreatic tumors revealed that pancreatic extracellular matrix contains a large amount of Mg^2+^ due to the leakage of pancreatic juice which promotes the formation of collagen type-I^45^. Our results *in vivo* suggest that this event should have occurred in order to justify similar results. In addition, after the implantation of Mg^2+^ tablets, it was observed numerous fragmentations in tablets and invagination of collagen type-III fibers in the spaces between the fragments, a fact that may be due to the mechanical weakness of magnesium-aluminum LDH tablet or the influence of magnesium LDH physicochemical properties on biological response. These two points could promote a rapid induction of collagen formation and subsequent invagination trough spaces between fragments. Similar feature was reported *in vitro* study in which the presence of MgCl_2_ on the mica surface promoted the disorderly assembling of collagen fibers on the inorganic substrate^44^. The fragmentation of Mg_2_Al-Cl tablet can also be related to a higher solubility of magnesium–aluminum LDH compared to zinc-aluminum material, as it was observed in experiments *in vitro* about the release of sulindac drug intercalated into LDHs in buffer solution pH 7.2 and 37 °C^46^.

Zn_2_Al-Cl sample exhibited a predominance of type-III fibers or reticular fibers, which were organized in parallel around the tablets. In the healing process, besides the involvement of collagen fibers, it can be noticed the inclusion of other proteins present in the extracellular matrix, such as fibronectin. It is known that fibronectin has a zinc-dependence binding domain called *Gelatin-Binding Domain* (GBD) in its structure, which interact with the collagen molecules^47^. In the presence of a large amount of Zn^2+^, the GBD domain captures a greater quantity of zinc ions slowing down the formation of fibronectin protein. This process lead to the delay formation of mature collagen fibers which cause the fibers to clump linger. We believe that this event has occurred in this study since the green birefringence was observed, and the thickness of the fibers was thin, showing the typical characteristic of collagen type-III fibers. Another factor contributing to the occurrence of type III can be the result of zinc ion binding to collagen molecules, modifying their structure assembly. The interaction of Zn^2+^ with collagen is reported in the literature^48^,^49^. These events could justify the presence of collagen type-III fibers around Zn_2_Al-Cl tablets.

**Figure 1:**
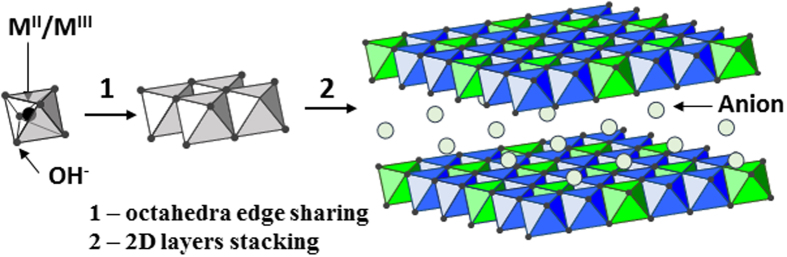
Schematic representation of the Layered Double Hydroxide structure.

**Figure 2:**
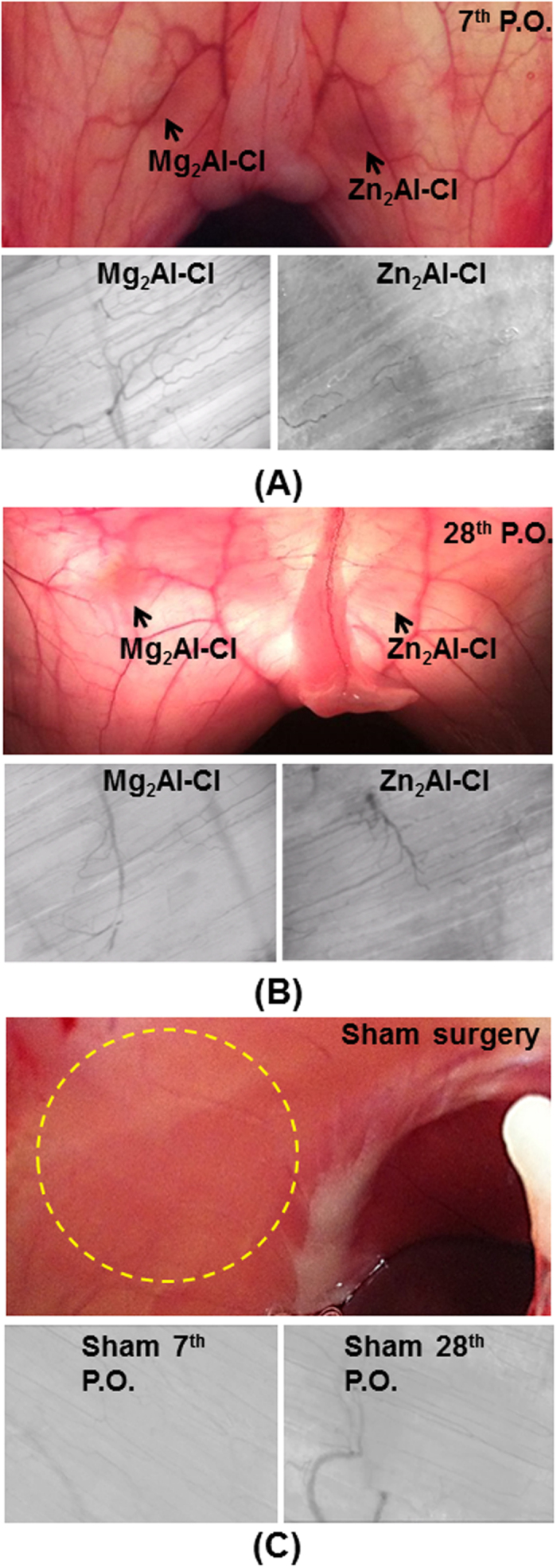
Colour photographs show the shadow of LDH tablets (black arrows) between muscle layers of the both sides of the abdominal wall, anatomically separated by xiphoidal appendix, while the grey pictures show the pattern of the microcirculation over the tablets, captured by SDF videomicroscopy “*in vivo*”, both at 7th (**A**) and 28th (**B**) P.O. days. The coloured images were taken from the peritoneal side, following a large U-shape incision of the abdominal wall and with aid of the transillumination. At both time periods, no signs of tissue inflammation were seen around tablets, and the microcirculatory network was also of normal aspect, showing a continuous blood flow without capillaries hemorrhage or thrombosis. SDF images size are of 1 mm^2^/frame containing muscle microvessels. Sham surgery: surgical procedures mimicking the tablet implantation procedure (**C**). *Superior* - Normal macroscopic aspect of the rat abdominal wall of the naïve animal, viewed from peritoneal side; circle indicates the usual site for the tablet implantation. *Inferior* - SDF images of the abdominal wall of the control group, showing normal pattern of microcirculatory at 7th and 28th P.O. days.

**Figure 3:**
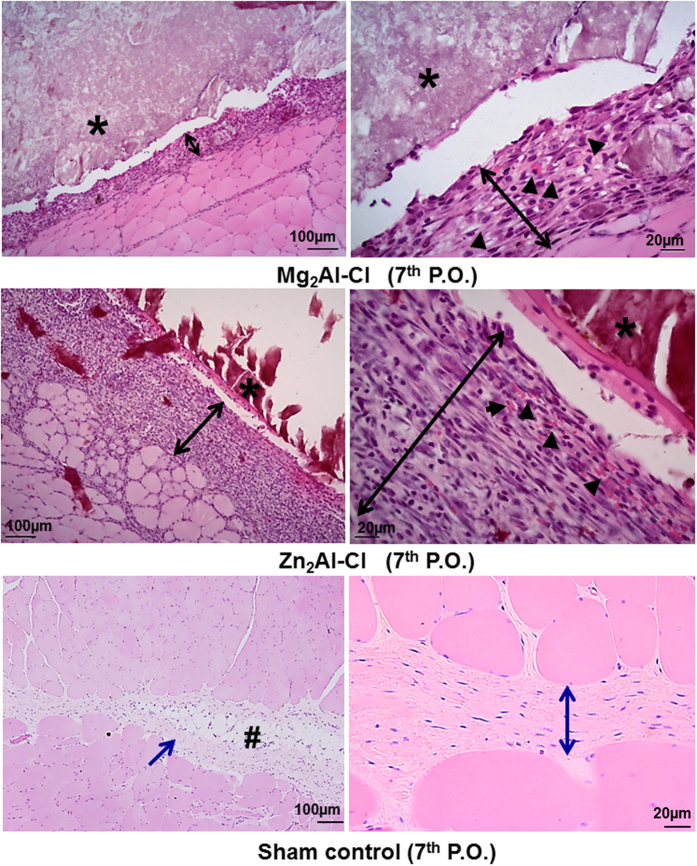
Histological results following Mg_2_Al-Cl LDH and Zn_2_Al-Cl LDH tablets implants between abdominal wall intermuscular spaces, showing their interaction with host cells after 7 days (7^th^ postoperative or P.O.). Inferior panel shows the normal histological aspect at 7^th^ P.O. after sham surgery. Images are shown in two magnifications to each sample. (*) LDH tablet; (↔black) new tissue; (►) neo-microvessels; (#) adipose tissue; (↔, →blue) connective tissue. Hematoxylin-eosin staining.

**Figure 4:**
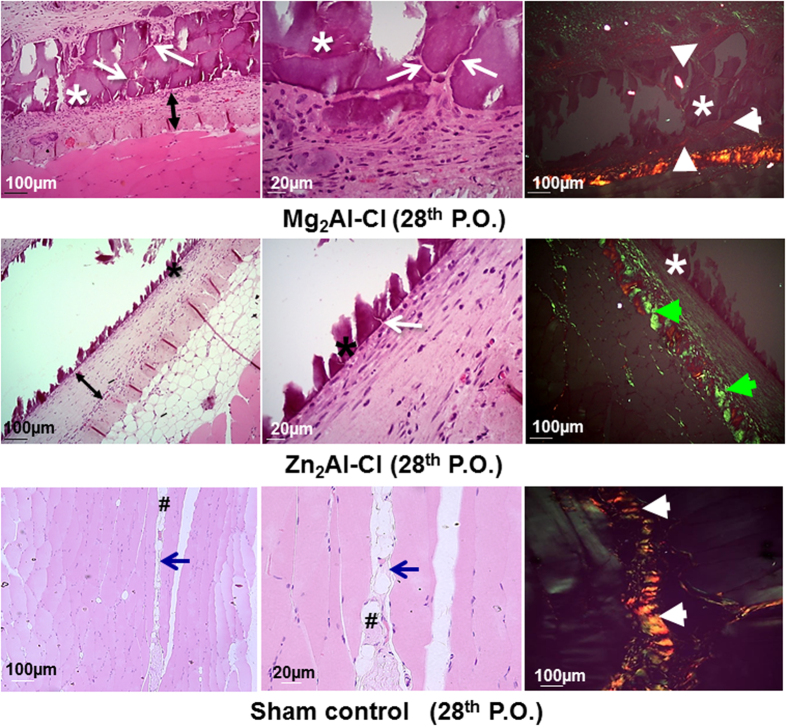
Histological aspect after 28 days of the implants showing the final tissue reconstruction and the persistence of implanted LDH tablets. Mg_2_Al-Cl (Superior row); Zn_2_Al-Cl (Middle row). Sham control group results (Inferior row) showed a normal aspect of tissue. (*) LDH tablet; (↔) new tissue; (→ white) collagen invagination; (► white) collagen type-I; (► green) collagen type –III; (→ blue) space between abdominal wall muscle layers; (#) adipose tissue. Hematoxylin-eosin (left and middle column) and Picrosirius red with birefringence (right column).

**Figure 5:**
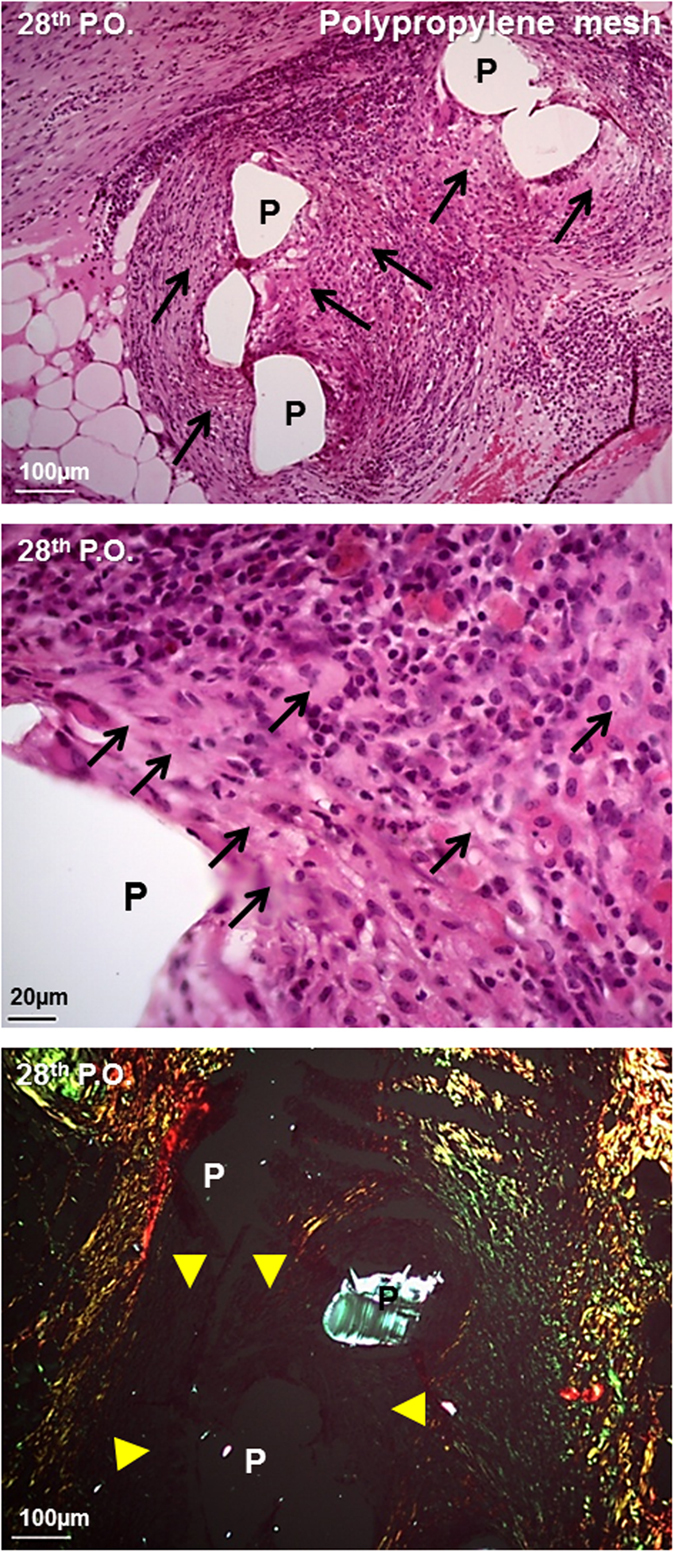
Histological evaluation of the abdominal wall surgical Polypropylene (P) mesh implants on nearby tissues stained with Hematoxylin-eosin (superior and middle) and Picrosirius red (inferior) after 28 days (28^th^ postoperative or P.O.). (→ black) multiple necrosis; (► yellow) edema and granulomatous reaction.

Collagens are extracellular proteins composed mainly by the amino acids glycine, proline, and 4-hydroxyproline^50^. Considering the structure and chemical compositions of LDHs, which comprise –OH groups at basal surfaces and edges (Fig. 1), these inorganic materials and residues positioned outside of collagen fibrils can interact through hydrogen bonds bridged or not by water molecules. The positive surface charge of LDH materials (+41.7 mV for Mg_2_Al-Cl and +46.0 mV for Zn_2_Al-Cl) plays an important role in the establishment of interactions between the inorganic material and biomolecules as proteins^2^. Although study about interactions involving LDHs and collagens is not reported in the literature, the net negative electric charge of residues in this protein should also drive interactions of electrostatic nature with LDHs.

The good tissue repair response observed around the LDH tablets may also be due to the alkaline pH buffering behavior of the LDHs. Certain factors promote an acidic extracellular pH value at sites of inflammation^51^. The acidic extracellular environment restricts cellular activities such as chemotaxis, respiratory activity and proliferative capacity of leukocytes, and consequently the response of tissue repair. Thus, maintenance of the alkaline extracellular pH value by the slowly dissolution of LDH tablets in the tissue may minimize local acidosis, promoting the preservation of normal cell responses.

## Conclusion

The overall histological results showed that LDH, regardless of their metals composition, triggers a good tissue repair with characteristic biocompatibility, promote the deposition of collagen and the appropriate extracellular matrix remodeling. At the same time, the results suggest that depending on the target effect, the LDH composition with either magnesium or zinc can be designed to influence the tissue remodeling to the desired collagen type. The biocompatibility, non-toxicity and non-immunogenic feature of LDHs verified in this study allow conjecturing their use as matrices for drug delivery associated or not to implantable devices.

## Additional Information

**How to cite this article**: Cunha, V. R. R. *et al.* Accessing the biocompatibility of layered double hydroxide by intramuscular implantation: histological and microcirculation evaluation. *Sci. Rep.* **6**, 30547; doi: 10.1038/srep30547 (2016).

## Supplementary Material

Supplementary Information

Supplementary Video S1

Supplementary Video S2

Supplementary Video S3

Supplementary Video S4

Supplementary Video S5

Supplementary Video S6
