# Supplementary material for: Accessing the biocompatibility of layered double hydroxide by intramuscular implantation: histological and microcirculation evaluation
Source: Sci Rep. 2016 Aug 2;6:30547. doi: 10.1038/srep30547 (PMC4969587; doi:10.1038/srep30547)
Supplement: Supplementary Information [file srep30547-s1.pdf]

SUPPLEMENTARY INFORMATION

Accessing the biocompatibility of layered double hydroxide by intramuscular implantation: histological and microcirculation evaluation

Vanessa Roberta Rodrigues Cunha[a], Rodrigo Barbosa de Souza[b], Ana Maria Cristina Rebello Pinto da Fonseca Martins[c], Ivan Hong Jun Koh[d,*], Vera Regina Leopoldo Constantino[a,**]

[a]*Departamento de Química Fundamental, Instituto de Química, Universidade de São Paulo - USP, Av. Prof. Lineu Prestes 748, CEP 05508-000, São Paulo, SP, Brazil.*

[b]*Departamento de Morfologia e Genética, Universidade Federal de São Paulo – UNIFESP, Rua Botucatu 740, CEP 04023-900, São Paulo, SP, Brazil.*

[c]*Instituto Biológico, Secretaria da Agricultura e Abastecimento, Av. Conselheiro Rodrigues Alves 1252, CEP 04014-002, São Paulo, SP, Brazil.*

[d]*Departamento de Cirurgia, Universidade Federal de São Paulo - UNIFESP, Rua Botucatu 740, CEP 04023-900, São Paulo, SP, Brazil.*

** Corresponding author. Departamento de Cirurgia, Universidade Federal de São Paulo - UNIFESP, Rua Botucatu 740, CEP 04023-900, São Paulo, SP, Brazil. ** Corresponding author. Departamento de Química Fundamental, Instituto de Química, Universidade de São Paulo - USP, Av. Prof. Lineu Prestes 748, CEP 05508-000, São Paulo, SP, Brazil.*

E-mail addresses: ivankoh@terra.com.br (I.H.J. Koh), vrlconst@iq.usp.br (V.R.L. Constantino).

LDH materials characterization

According to the metal content analysis and the weight percentage of H_2O (obtained from TGA curve), the chemical compositions of LDHs material prepared in this work are $[Mg_{2.10}Al(OH)_{6.20}]Cl\cdot 2.3H_2O$ and $[Zn_{2.08}Al(OH)_{6.16}]Cl\cdot 1.7H_2O$.

XRD patterns of chloride intercalated LDH samples are shown in Fig. S1. The basal spacing of the layered materials, corresponding to d_{003} value equal to 0.77 nm, is expected for a LDH containing intercalated chloride anions [1]. XRD patterns in the region up to 30° (2θ) show the diffraction peaks (012), (110) and (113) typical of the LDH structure [1]. Aqueous suspension of LDH samples are stable considering the high Zeta potential value observed: + 41.7 mV for Mg_2Al-Cl and + 46 mV for Zn_2Al-Cl. The medium hydrodynamic diameter of the magnesium and zinc LDHs particles is 85 nm and 104 nm, respectively.

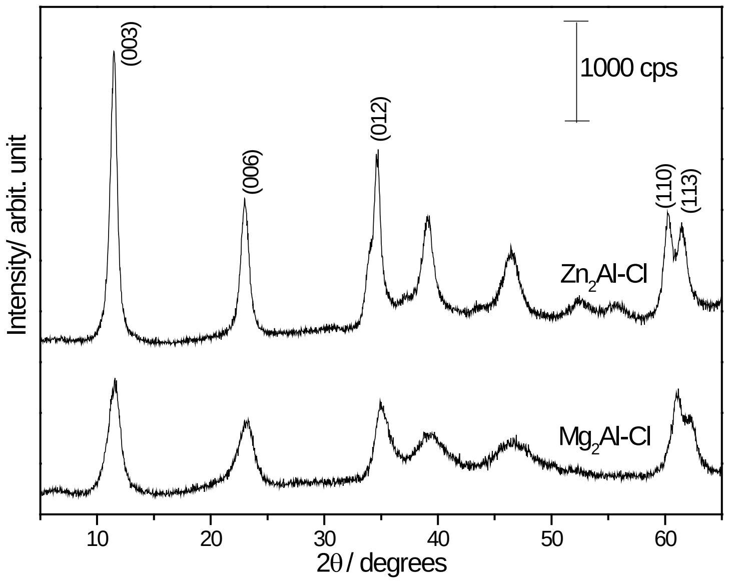

Figure S1 | XRD patterns of powdered Zn_2Al-Cl and Mg_2Al-Cl samples.

FT-IR spectra of the LDH-Cl samples (Fig. S2, black line) present the broad band at 3550 cm^{-1} assigned to O-H stretching of the extrinsic (adsorbed on the layer) and intrinsic (interlayer) water molecules and also the hydroxyl groups of the layers. The absorption band at 1631 cm^{-1} is attributed to the bending of water molecules while the broad band below 1000 cm^{-1} is assigned to metal-OH translation modes of LDH layers [2]. The vibrational mode related to the symmetric stretching of the carbonate anion is active in the Raman spectra and occurs at 1061 cm^{-1} (Fig. S2, blue line). This band can be observed in the spectra of both LDH-Cl samples (Figs. S2A and S2B) but with very low intensity for Zn_2Al-Cl (Fig. S2B). Therefore, the amount of carbonate anions in the samples is insignificant. The band at 540-560 cm^{-1} in the FT-Raman spectra is related to the vibrational mode of the metal-OH in the layers.

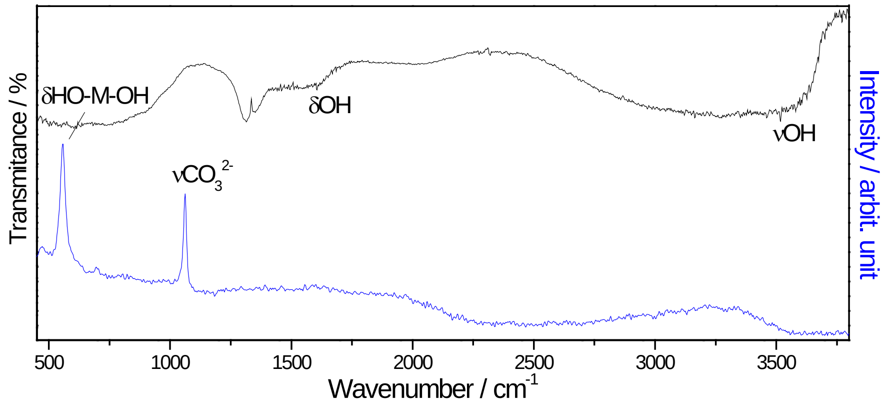

(A)

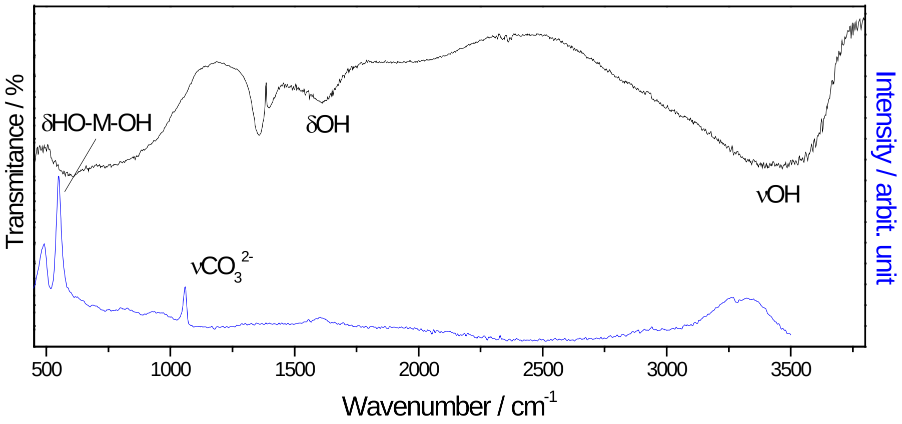

(B)

Figure S2 | FT-IR (black) and FT-Raman (blue) spectra of Mg_2Al-Cl (A) and Zn_2Al-Cl (B) samples.

Thermal analysis data of Mg_2Al-Cl and Zn_2Al-Cl samples (Figs. S3A and S3B, respectively) are in agreement with the expected for the LDH materials [3]. The weight losses occur in the following temperature ranges for Mg_2Al-Cl: 25 to 220 °C (1° event); 320 to 450 °C (2° event) and above 450 °C (3° event). On the other hand, it is observed the following events to Zn_2Al-Cl: 25 to 150 °C (1° event); 150 to 330 °C (2° event) and above 400 °C (3° event). The temperature range of each event was determined by DTG curves. The first event is attributed to the samples dehydration, *i.e.*, the release of water molecules superficially bonded to the layers followed by the interlayer ones (MS curves show the delivery of fragment with *m/z* equal to 18). This process corresponds to 16.2 % to Mg_2Al-Cl and 9.1 % to Zn_2Al-Cl. The second event correspond to the simultaneous release of species with *m/z* equal to 18 (H_2O from layers dehydroxylation) and 44 in minor proportion (fragment related to CO_2 from the carbonate presence). The third event, which corresponds to temperatures above 450 °C for Mg_2Al-Cl and 400 °C for Zn_2Al-Cl, occurs the release of HCl (dehydrochlorination; *m/z* fragment equal to 36) and the irreversible formation of MO and MAl_2O_4, where M can be whether Mg^{2+} or Zn^{2+} (spinel phase) [4].

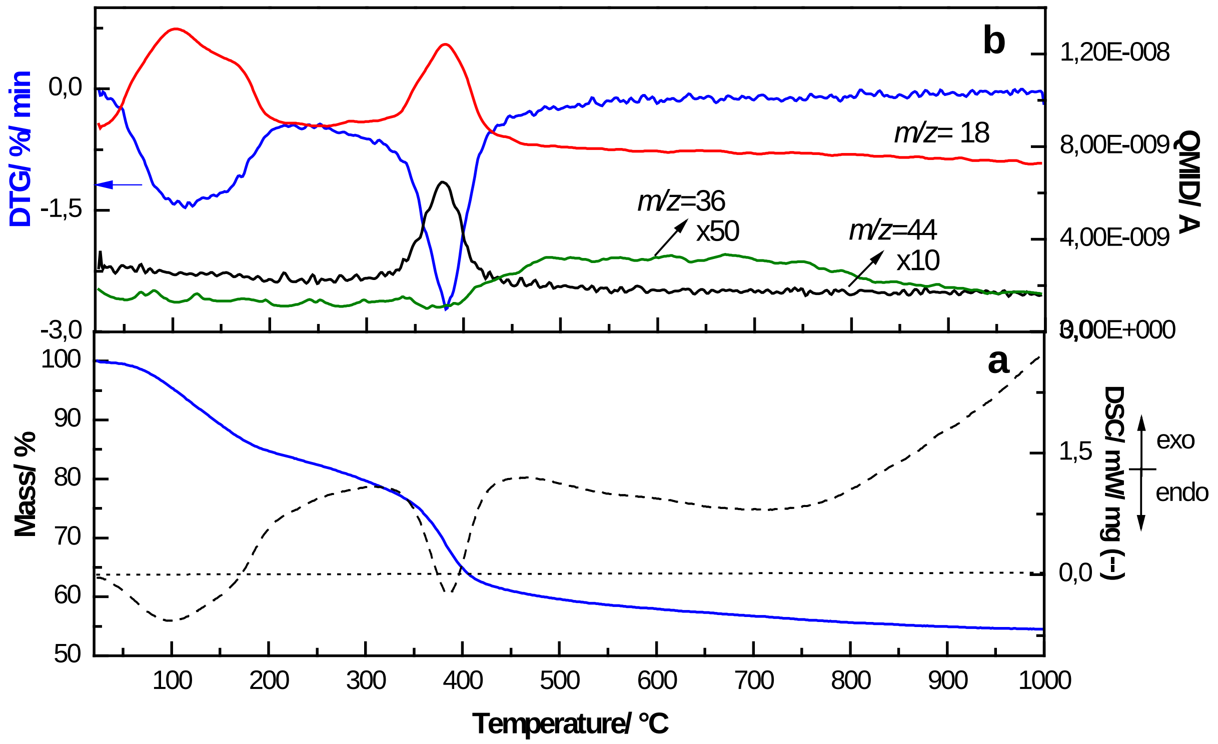

(A)

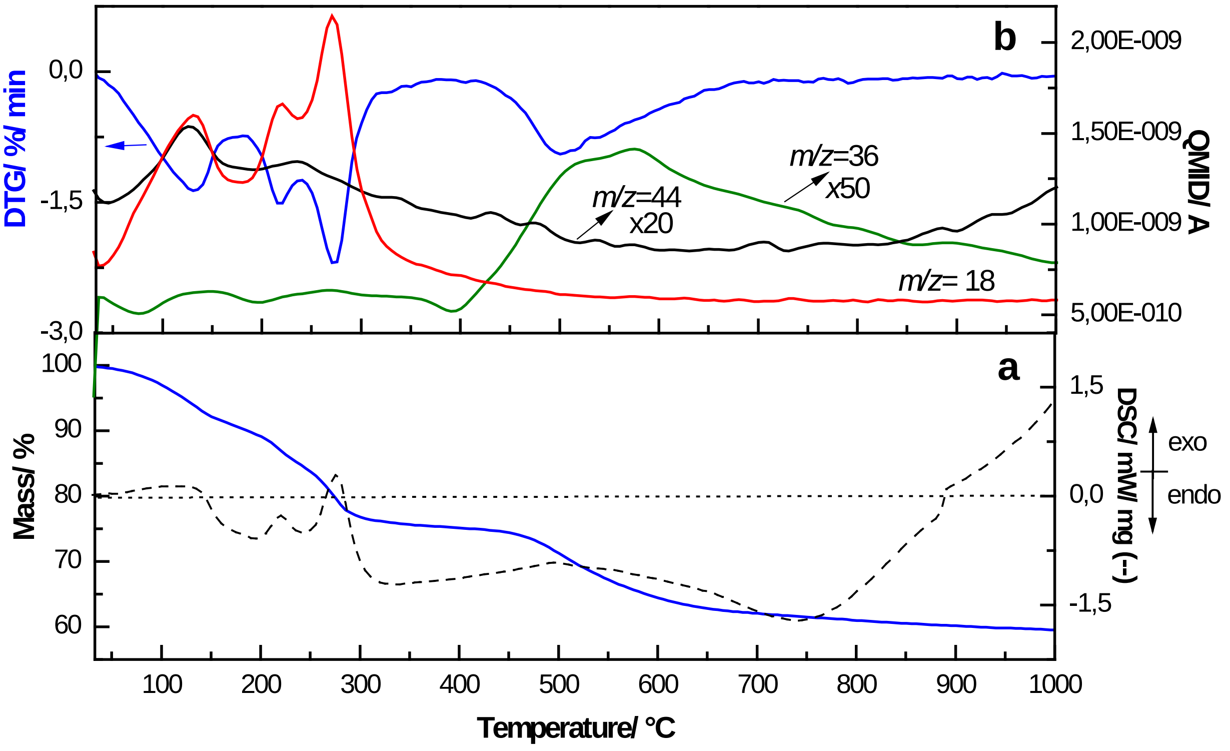

(B)

Figure S3 | TGA (blue) – DSC (black) (a); and DTG (blue) - MS (black, red and green) (b) curves of (A) Mg_2Al-Cl and (B) Zn_2Al-Cl samples.

Sidestream Dark Field Assessment

Examination by video microscopy (SDF), Videos S1 to S4, revealed an overall integrity of the microcirculatory network in muscle tissues involving the tablets, with continuous blood flow. Therefore, no obstruction, bleeding and/or increasing of leukocyte-endothelial adhesion were noticed. Such factors are normally present in microvessels exposed to nearby antigenic material implants. SDF results of the control group (Sham surgery) are shown in Videos S5 and S6.

Video S1. The microcirculatory network of the Mg_2Al-Cl tablet examined after 7 days, captured by SDF video microscopy "*in vivo*" over the LDH tablet (black and white images), presented normal aspect and a continuous blood flow at both times of study. SDF images are of 1 mm^2 /frame containing muscle microvessels.

Video S2. The microcirculatory network of the Mg_2Al-Cl tablet examined after 28 days, captured by SDF video microscopy "*in vivo*" over the LDH tablet (black and white images), presented normal aspect and a continuous blood flow at both times of study. SDF images are of 1 mm^2 /frame containing muscle microvessels.

Video S3. The microcirculatory network of the Zn_2Al-Cl tablet examined after 7 days, captured by SDF video microscopy "*in vivo*" over the LDH tablet (black and white images), presented normal aspect and a continuous blood flow at both times of study. SDF images are of 1 mm^2 /frame containing muscle microvessels.

Video S4. The microcirculatory network of the Zn_2Al-Cl tablet examined after 28 days, captured by SDF video microscopy "*in vivo*" over the LDH tablet (black and white images), presented normal aspect and a continuous blood flow at both times of study. SDF images are of 1 mm^2 /frame containing muscle microvessels.

Video S5. The microcirculatory network of the control group (Sham surgery) examined after 7 days, captured by SDF video microscopy "*in vivo*", presented normal aspect and a continuous blood flow. SDF images are of 1 mm^2 /frame containing muscle microvessels.

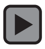

Video S6. The microcirculatory network of the control group (Sham surgery) examined after 28 days, captured by SDF video microscopy "*in vivo*", presented normal aspect and a continuous blood flow. SDF images are of 1 mm^2 /frame containing muscle microvessels.

References

[1] Cunha, V. R. R. *et al.* Structural, Spectroscopic (NMR, IR, and Raman), and DFT Investigation of the Self-Assembled Nanostructure of Pravastatin- LDH (Layered Double Hydroxides) Systems. *Chem. Mater.* **24**, 1415-1425 (2012).

[2] Kloprogge, J.T., Hickey, L. & Frost, R.L. FT-Raman and FT-IR spectroscopic study of synthetic Mg/Zn/Al-hydrotalcites. *J. Raman Spectrosc.* **35**, 967-974 (2004).

[3] Yun, S.K. & Pinnavaia, T.J. Water Content and Particle Texture of Synthetic Hydrotalcite-like Layered Double Hydroxides. *Chem. Mater.* **7**, 348–354 (1995).

[4] Belloto, M., Rebours, B., Clause, O., Lynch, J., Bazin, D. & Elkaim, E. Hydrotalcite Decomposition Mechanism: A Clue to the Structure and Reactivity of Spinel-like Mixed Oxides. *J. Phys. Chem.* **100**, 8535-8542 (1996).
